# Steroid Cell Tumor of the Ovary Presenting With Postmenopausal Bleeding and Hyperandrogenism: A Case Report

**DOI:** 10.7759/cureus.111421

**Published:** 2026-06-24

**Authors:** Supreet Mahurkar, Sumi Surendran, Jaya Cherian

**Affiliations:** 1 Obstetrics and Gynaecology, Aneurin Bevan University Health Board, Gwent, GBR

**Keywords:** hirsutism, hyperandrogenism, ovarian neoplasm, postmenopausal bleeding, steroid cell tumor

## Abstract

Steroid cell tumors of the ovary are rare, often hormonally active neoplasms that may present with features of hyperandrogenism. We report the case of a 60-year-old woman who initially presented with postmenopausal bleeding and a reassuringly thin endometrium, with symptoms attributed to hormone replacement therapy. Over time, the development of progressive hirsutism and persistently elevated serum testosterone levels prompted further investigation. Multidisciplinary team involvement, including endocrinology input, was pivotal in identifying a suspected androgen-secreting ovarian tumor and guiding definitive management. Bilateral salpingo-oophorectomy confirmed a steroid cell tumor of the left ovary, International Federation of Gynecology and Obstetrics (FIGO) stage IA, with complete biochemical and clinical resolution. This case highlights the importance of reassessing postmenopausal patients with evolving hyperandrogenic features despite initially reassuring findings.

## Introduction

Steroid cell tumors (SCTs) of the ovary are rare sex cord-stromal neoplasms characterized by steroid hormone production, most commonly androgens [1]. They account for fewer than 0.1% of all ovarian tumors and are most frequently classified as SCTs not otherwise specified (NOS) [1,2]. Although the majority are benign, up to 25-40% may exhibit malignant behavior [1,2].

The hormonal activity of SCTs can result in a range of clinical manifestations, including hirsutism, virilization, menstrual irregularities, and postmenopausal bleeding [3,4]. In postmenopausal women, physiological androgen production is minimal; therefore, new-onset hyperandrogenism should prompt thorough evaluation for an underlying ovarian or adrenal source [5,6]. Ovarian neoplasms are staged according to the International Federation of Gynecology and Obstetrics (FIGO) classification system [7].

This case illustrates how an initially reassuring presentation of postmenopausal bleeding can evolve into the diagnosis of a rare androgen-secreting ovarian tumor, with progressive hirsutism serving as a key diagnostic clue.

## Case presentation

A 60-year-old multiparous woman (P4, four previous spontaneous vaginal deliveries) was referred to the gynaecology service with intermittent postmenopausal bleeding occurring every few weeks. Her medical history included endometriosis and uterine fibroids. She had previously undergone hysteroscopy with the insertion of a levonorgestrel intrauterine system (Mirena®, Bayer plc, Reading, UK), which had been removed approximately two years before presentation. She had been amenorrhoeic while using the device, making her menopausal status uncertain. Cervical screening was up to date and normal.

At the initial assessment, transvaginal ultrasound demonstrated a thin endometrium measuring 2.7 mm, with the right ovary not visualized and a normal-appearing left ovary. An endometrial biopsy was attempted but yielded an inadequate sample. A Mirena® device was reinserted, and hormone replacement therapy was planned.

Seven months after the initial presentation, the patient re-presented with continuous vaginal bleeding while receiving hormone replacement therapy. Oestrogen therapy was discontinued, after which the bleeding resolved.

Twelve months after the initial presentation, she reported occasional postcoital bleeding and progressive hirsutism that had developed over several months. Hormonal evaluation demonstrated postmenopausal gonadotropin levels with elevated serum testosterone. Serial testing showed a progressive rise in testosterone from 4.5 nmol/L to 8.1 nmol/L. The hormonal profile is summarized in Table 1.

**Table 1:** Hormonal profile demonstrating elevated serum testosterone with postmenopausal gonadotropin levels

Parameter	Value	Reference range (postmenopausal female)
Follicle-stimulating hormone (FSH) (IU/L)	115.7	23-116.3
Luteinizing hormone (LH) (IU/L)	52.2	7.9-53.8
Oestradiol (pmol/L)	110	<118
Progesterone (nmol/L)	2	3-5
Testosterone (nmol/L)	4.5 → 8.1	0.3-1.3
Sex hormone-binding globulin (nmol/L)	64	24-111
Free androgen index (%)	7	0.3-2.5

**Figure 1:**
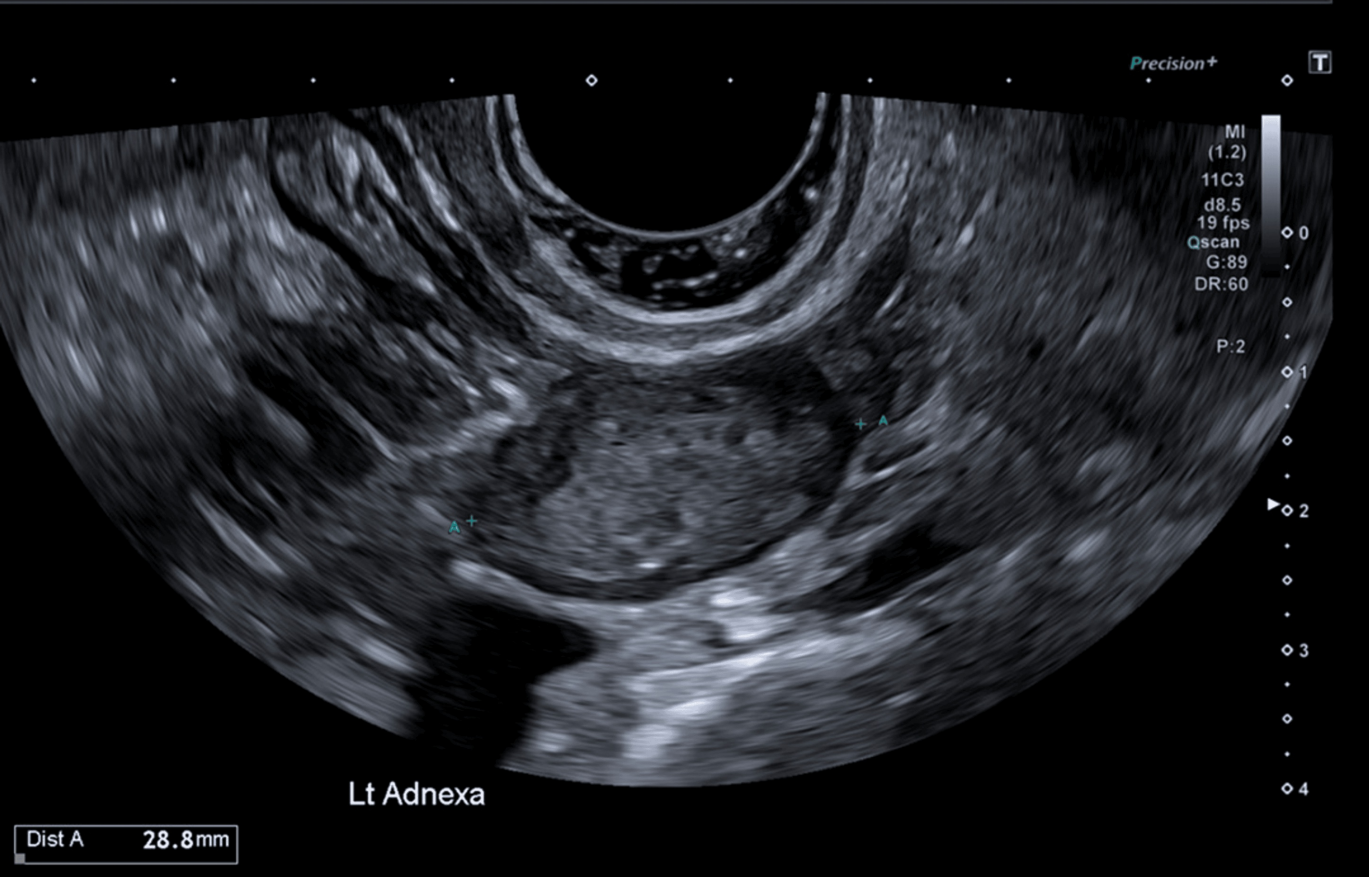
Transvaginal ultrasound demonstrating a homogeneous solid echogenic mass in the left adnexa measuring approximately 2.7×1.8×2.9 cm

Sixteen months after the initial presentation, pelvic ultrasound revealed a homogeneous solid echogenic mass measuring approximately 2.7×1.8×2.9 cm in the left adnexa (Figure 1). Repeat ultrasound confirmed a solid lesion adjacent to a normal-appearing ovary, with displacement of the Mirena® device, which was subsequently removed. Magnetic resonance imaging (MRI) demonstrated a bulky left ovary with an indeterminate solid lesion.

Tumor markers were within normal limits, including cancer antigen 125 (CA-125) (19 kU/L), alpha-fetoprotein (6 kU/L), carcinoembryonic antigen (CEA) (<2 µg/L), and carbohydrate antigen 19-9 (CA 19-9) (<4 kU/L).

The case was discussed in a multidisciplinary team meeting. Endocrinology input raised a strong suspicion for an androgen-secreting ovarian tumor based on the biochemical profile and clinical progression, and surgical management was recommended.

Twenty-four months after the initial presentation, the patient underwent laparoscopy, bilateral salpingo-oophorectomy, hysteroscopy, and resection of a submucosal fibroid. Histopathological examination demonstrated an 18 mm SCT of the left ovary, without capsular disruption or surface involvement, consistent with FIGO stage IA disease.

Postoperatively, serum testosterone normalized to 0.4 nmol/L, with the complete resolution of hyperandrogenic symptoms. A staging computed tomography (CT) scan of the thorax, abdomen, and pelvis showed no evidence of metastatic disease. Following a multidisciplinary team discussion, she subsequently underwent total laparoscopic hysterectomy with omental biopsy, with no evidence of residual malignancy on final histology. She remains under routine gynaecological surveillance.

## Discussion

This case highlights the diagnostic complexity of SCTs of the ovary, particularly when early findings appear reassuring. The initial presentation with postmenopausal bleeding understandably focused attention on uterine pathology. A reassuringly thin endometrial thickness on ultrasound, combined with subsequent hormone replacement therapy use, led to the assumption that persistent bleeding was treatment‑related.

Resolution of bleeding after the cessation of oestrogen therapy further supported this assumption. However, the later development of progressive hirsutism proved to be a crucial diagnostic turning point. New‑onset hyperandrogenism in postmenopausal women is abnormal and should prompt evaluation for androgen‑secreting tumors, irrespective of prior reassuring imaging findings [5,6].

SCTs are often small and radiologically indeterminate yet can be disproportionately hormonally active [1,3]. Serial biochemical monitoring demonstrating progressively rising testosterone levels was instrumental in identifying an ovarian source, consistent with published recommendations for evaluating postmenopausal hyperandrogenism [5,6].

Multidisciplinary collaboration, particularly with the endocrinology team, played a pivotal role in guiding management. Bilateral salpingo‑oophorectomy served both diagnostic and therapeutic purposes, with immediate normalization of androgen levels, as demonstrated in previous reports [3,8]. Although histological features associated with malignancy, such as large tumor size, capsular invasion, necrosis, or increased mitotic activity, were absent [2], continued follow‑up remains appropriate due to reported cases of late recurrence [1].

## Conclusions

SCTs of the ovary are rare but important causes of hyperandrogenism in postmenopausal women. This case demonstrates how reassuring early findings and attribution of symptoms to hormone replacement therapy may delay diagnosis. The development of hirsutism should prompt reassessment and further investigation. Early multidisciplinary involvement and timely surgical intervention lead to excellent outcomes.
